# 3D Molding of Veneers by Mechanical and Pneumatic Methods

**DOI:** 10.3390/ma10030321

**Published:** 2017-03-22

**Authors:** Milan Gaff, Miroslav Gašparík

**Affiliations:** Department of Wood Processing, Faculty of Forestry and Wood Sciences, Czech University of Life Sciences in Prague, Kamýcká 1176, Prague 6–Suchdol 16521, Czech Republic; gathiss@gmail.com

**Keywords:** 3D molding, veneer, maximum deflection, lamination, plasticizing, ammonia

## Abstract

This paper deals with the influence of selected methods (mechanical and pneumatic) as well as various factors (wood species, moisture content, veneer shape, punch diameter, laminating foil thickness, holding method, plasticizing) on 3D molding of veneers. 3D molding was evaluated on the basis of maximum deflection of birch and beech veneers. Cracks and warping edges were also evaluated in selected groups of mechanical molding. Mechanical methods tested veneers with various treatments (steaming, water and ammonia plasticizing and lamination). The pneumatic method was based on veneer shaping using air pressure. The results indicate that birch veneers are more suitable for 3D molding. The differences between the mechanical and pneumatic methods were not considerable. The most suitable method for mechanical 3D molding was the veneer lamination by polyethylene foils with thicknesses of 80 and 125 μm, inasmuch as these achieved better results than veneer plasticized by steam. The occurrence of cracks was more frequent in beech veneers, whereas, edge warping occurred at similar rates for both wood species and depends rather on holding method during 3D molding. Use of the ammonia solution is more suitable and there occurs no marked increase in moisture as happens when soaking in water.

## 1. Introduction

New and unusual shapes of furniture parts are forcing designers and technologists still to look for new methods of material molding. For this reason, the manufacturers endeavor to emphasize functionality of the products as well as design technologies for manufacturing these products with the highest possible productivity, lowest costs, and minimal time requirements.

Three-dimensional molding of planar components is one of the most complicated chipless methods of shaping wood. According to Wagenführ and Buchelt [1], three-dimensional veneer molding, in contrast to molding of plastics and certain other materials, has limited application, which stems from the properties of wood, specifically from its low tensile deformation, small plastic deformation, and anisotropic properties.

**Figure 1:**
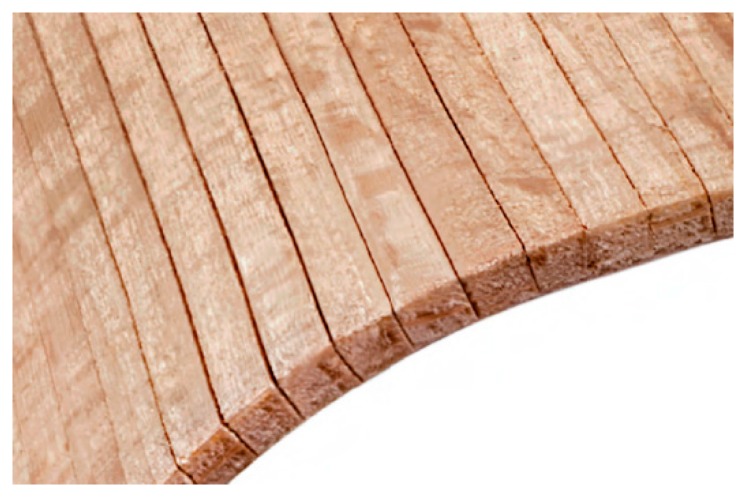
Basic veneer created on the Danzer principle. Adapted from [2], with permission from © 2016 Danzer Veneer Europe GmbH.

A representative organization dealing with this topic internationally is Danzer Veneer Europe GmbH [2]. This company has developed a method based on the cutting of 1.1–1.2 mm-thick veneers lengthwise, parallel to the grain direction, into strips 1-mm wide. Subsequently, these strips were pressed together and joined using polyamide fiber (Figure 1) [2]. Joining has to be carried out immediately after the veneers are cut in order to prevent the veneer strips from shifting longitudinally. Thusly, shaped veneer layers are used as the basic veneer for the internal layer of the future composition of a 3D veneer.

**Figure 2:**
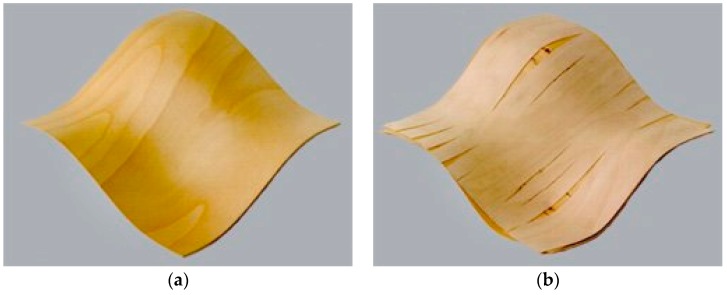
3D molding of veneer: (**a**) 3D veneer; (**b**) conventional plywood. Adapted from [2], with permission from © 2016 Danzer Veneer Europe GmbH.

Modified and non-modified veneers are stacked into a multi-layer set similarly to plywood but without cross-banding. The glue is spread on the veneers, which are then pressed using a mold. This modification technique prevents increasing transversal extensibility due to which there occur no longitudinal cracks as in non-modified veneer products (e.g., plywood) (Figure 2) [2]. Nevertheless, even this method is not perfect and the deflection of the bends, that can be achieved, is still limited. Other disadvantages are this method’s technological demandingness and its destructive nature.

**Figure 3:**
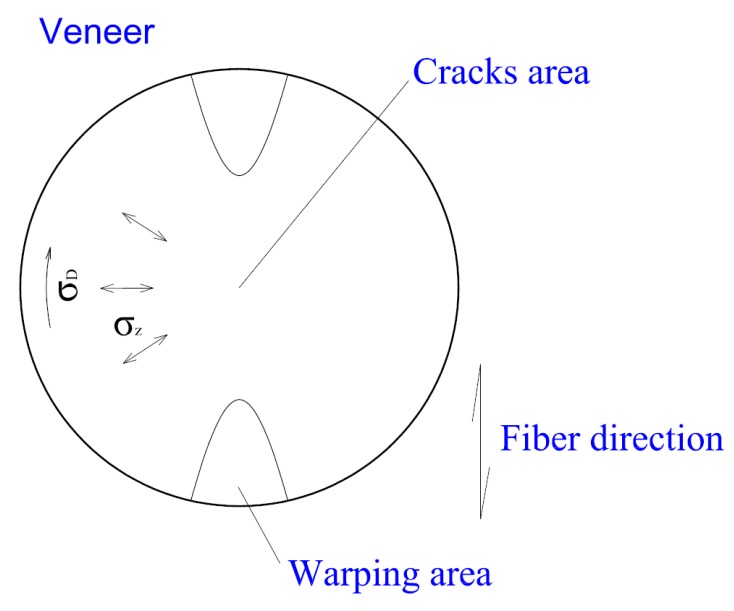
Representation of emerging stresses in the veneer and possible locations with defects.

In 3D molding, pressure acts tangentially and tension radially on the veneer, thus causing edge warping and cracks further inward from the edges of the veneer (Figure 3). Warping as well as cracks has to be evaluated as defects in 3D molding.

Warping occurs in the plane perpendicular to the direction of compression. If the veneer has a small thickness, the compression does not warp the veneer. In thicker veneers there occur cracks further inward from the edges of the sample. The cracks are caused by radial tensions exceeding the tension resistance across the grain [3]. Wagenführ [3] stated that the warping, which occurs at the edge of the veneers, can be eliminated by clamping of the veneer in special jig during bending. Suppression of the influence of the planar shape and veneer warping at the edges can be achieved also by ensuring an equal distance of each point of the sample’s circumference from its center.

Establishing suitable conditions can effectively modify the veneers’ properties and increase their 3D moldability while maintaining the qualitative and especially aesthetic parameters of the final products. In addition to the aforementioned method of holding, the veneer deflection achieved during 3D molding is influenced by other factors, such as wood species, sample shape, molding tool shape, and wood moisture. Wood species with long grains, uniform annual rings, and good plasticizing ability are most suitable for bending. For this reason, deciduous wood species (e.g., beech and birch) are more suitable for bending than are coniferous wood species.

Plasticizing is a traditional type of wood treatment using the effects of moisture and heat on wood, and it is performed prior to the bending itself [4]. The main objective of wood plasticizing is temporarily to change the physical and mechanical properties to create optimal conditions for forming while simultaneously minimizing damage to components of the lignin–saccharide matrix as reported by Peres et al. [5]. Bending characteristics of wood are affected the most by moisture in association with higher temperature as recently stated by Kutnar and Kamke [6]. All mechanical properties decrease with increasing moisture content from zero to the fiber saturation point (FSP) [7], and this makes forming easier. Achieving a desirable state depends also on the method of plasticizing as stated by Gašparík and Barcík [8].

Currently, there are several methods of plasticizing: hydrothermal, electromagnetic, and chemical [4]. The first two of these methods are used predominantly. Chemical methods are applied mainly in densification (pressing) of wood and in creating extreme shapes or complicated structures without diminishing strength (e.g., surface embossing) [9]. Traditional wood steaming is one of the oldest and most widely used types of plasticizing. The advantages of this method include good wood plasticity and only slight degradation caused by longer plasticizing times. Disadvantages of steaming include longer process times, slow permeation of heat from the surface to the center, and higher wood moisture after plasticizing. Plasticizing using ammonia (liquefied or gaseous ammonia or an aqueous solution) is among the important chemical methods [10]. Wood modification using liquefied and gaseous ammonia is technically demanding and requires a special apparatus [11]. An easier way is to treat wood with an aqueous solution of ammonia, but this usually is accompanied by an undesirable increase in wood moisture. Increased wood moisture is not very suitable for practical application because the veneer-shaping process is usually associated with gluing. The gluing process can be performed only after subsequent moisture adjustment.

Another known method used in wood shaping, complementing the plasticizing effect and ensuring increase in bendability values, is the well-known use of a flexible metal strap, sometimes referred to as bending strap. It has been demonstrated both theoretically and in practice that even if wood is plasticized, damage occurs mainly in the tension zone. This shortcoming can be prevented by placing the bending strap on the convex side of the bent wood and securing the wood with the bending strap’s end stops [12]. In using a bending strap with end stops, the neutral line is shifted toward the strap and the cracks on the tension side are eliminated.

The objective of the present study is to identify the effects of selected factors influencing the maximum deflection of veneers in their 3D shaping using various methods. Beech and birch samples of circular and square shape, at moisture from 0% to wet were tested mechanically and pneumatically along with various treatments (plasticizing with water, steam, and ammonia), punch diameters, and types of holding.

## 2. Experimental Section

### 2.1. Material

Three-dimensional moldability was examined on square (60 mm × 60 mm, 80 mm × 80 mm, and 100 mm × 100 mm) and circular (ø 60, ø 80, and ø 100 mm) samples made of radial veneers of European beech (*Fagus sylvatica* L.) and Silver birch (*Betula pendula* Roth.) with an average thickness of 0.55 mm.

**Figure 4:**
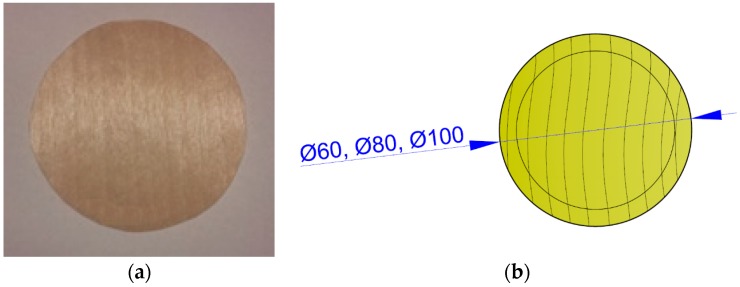
Sample with circular shape: (**a**) Real image of sample; (**b**) Drawn image of sample (unit: mm).

The samples were cut out of veneer sheets and did not contain any defects. A guide circle was drawn on the surface of the circular sample (Figure 4) used for centering the test sample. The circular shape of the test sample (Table 1) follows from the requirement that each point of the sample’s circumference be at an equal distance from its center.

**Figure 5:**
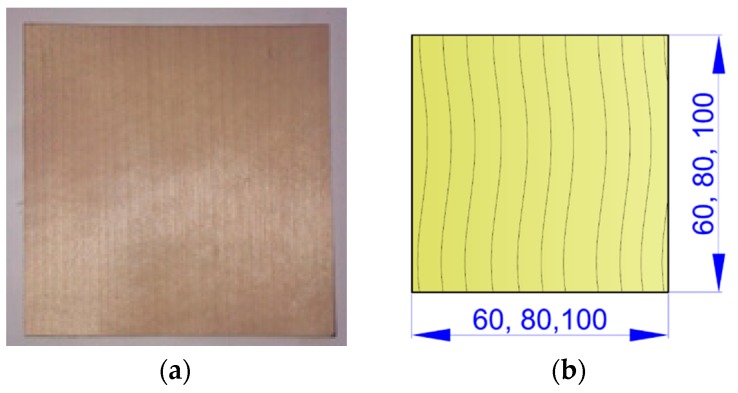
Sample with square shape: (**a**) Real image of sample; (**b**) Drawn image of sample (unit: mm).

The square shape of the other sample (Figure 5) was selected for two reasons. The first is that, in view of the technology used, a square shape of the components is more suitable for the given purpose (i.e., it is the natural shape the veneer has after manufacturing by slicing or peeling). In addition, this shape was used to verify the hypothesis of suppressing the influence of the sample’s planar shape on creation of veneer warping in some parts of its edge zone during shaping.

Samples were conditioned, for final moisture content from 8% to 30%, in a conditioning chamber KBF 115 (Binder, Tuttlingen, Germany) under conditions shown in Table 2. Oven-dry samples were prepared in the laboratory dryer FP 115 (Binder, Tuttlingen, Germany) at 103 ± 2 °C. Wet samples were soaked in water to reach maximum moisture content. Wood moisture content was determined according to ISO 13061-1 [13].

### 2.2. Methods

Table 3 summarizes all types of mechanical and pneumatic 3D molding as well as factors and conditions for the individual methods used in testing.

#### 2.2.1. General Basic Mechanical Method

**Figure 6:**
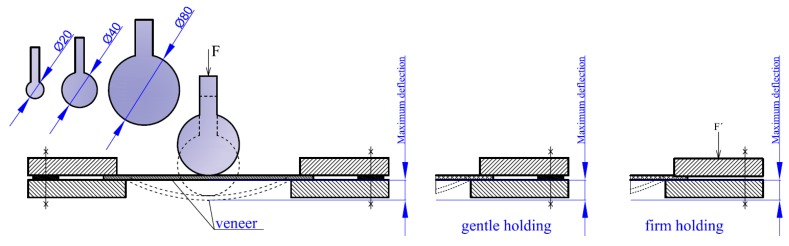
Schematic representation of a mechanical method (unit: mm).

The general basic method consisted of a 3D molding by mechanical loading (Figure 6), and that formed the basis for other variants of the mechanical method. Currently, there is no standardized method for evaluating 3D veneer moldability. Therefore, the Erichsen’s method used for evaluating moldability of sheet metal according to EN ISO 20842 [14] was modified for our purposes.

In this case, the three-dimensional moldability was assessed on beech and birch samples with square and circular shapes (Figure 4 and Figure 5) conditioned to moisture of 0%, 8%, 16%, 20%, 30%, and 100%. The samples were stressed with spherical punches of three different diameters: 20 mm, 40 mm, and 80 mm (Figure 6). The diameter of the internal opening through which the samples were shaped is 4 mm larger than the diameter of the punch. Therefore, openings with diameters of 24 mm, 44 mm, and 84 mm for the respective punches were used. Three-dimensional moldability was evaluated by maximum deflection (Figure 6). The holding system was developed in order to prevent the occurrence of warping at the circumference of the sample, which is held during the molding by the circumferential compressive force F’ (Figure 6). In this case, the samples were held with gentle holding. Gentle holding was achieved using a distance (washer) spacer, which was approximately 10% thicker than the veneer, thus allowing for a certain horizontal movement of the veneer during loading. Compression force that acted on the sample via the spherical punch was carried out using a universal testing machine LabTest 4.050 (LaborTech, Opava, Czech Republic) with a loading speed of 10 mm/min.

#### 2.2.2. Mechanical Method with Lamination Treatment and Steaming

The second method of 3D molding was also based on mechanical loading and with the same principle as in the previous case, but only on circular samples at 16% moisture. This method was tested also on beech and birch samples (veneers). The difference was in treatment of the samples, which were divided into three groups. The first was a reference group without any treatment. The second group was intended for treatment by laminating and the final group by steam plasticizing. Samples were loaded using spherical punches with diameters of 20 mm and 40 mm. In this case, pressure was applied with firm as well as gentle holding of the samples. In gentle holding of samples, the veneer has a certain horizontal movement due to the distance spacer, which was approximately 10% thicker than the veneer. In case of firm holding, in contrast, the edge of the veneer was firmly clamped by the flanges. This method also evaluated the defects that occurred (cracks and edge warping) using the X-Loupe Solution software.

**Figure 7:**
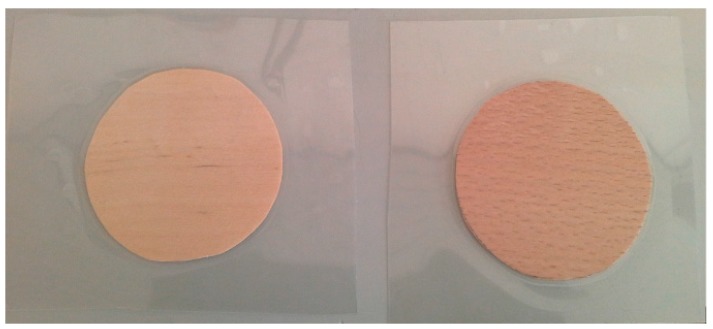
Veneer samples after lamination.

Laminated samples (Figure 7) were prepared by laminating with three different types of foil having thicknesses of 80-µm, 100-µm, and 125-µm produced by Peach^®^, Schindellegi, Switzerland. The laminating foils consisted of three layers: polyethylene terephthalate (PET), polyethylene (PE), and ethylene vinyl acetate (EVA) in the ratios 6:1.6:2.4. The purpose of laminating the samples was to change the pretension level and shift the neutral level into the laminating foil. Foil actually substitutes the support function of the bending strap with end stops derived from traditional wood bending.

Steam plasticizing was carried out in a plasticizing apparatus designed for this research. The water in the plasticizing apparatus was first preheated to 90 °C and the samples were then inserted. Plasticizing of the veneer samples was performed at 95 °C for 15 min.

#### 2.2.3. Mechanical Method with Plasticizing by Water and Ammonia

Another 3D molding method was based on mechanical loading following the same principle as in the previous case (circular samples with 16% moisture) but only on beech samples. The difference was in the plasticizing of the samples, which were divided into three groups. The first was a reference group with no treatment. The second group was plasticized with water, while the third group was plasticized by ammonia. The samples were loaded by punch with a 40 mm diameter. In this case, only firm holding of the samples was used.

Plasticizing with water was carried out by soaking samples in water at temperatures of 20 °C and 95 °C for 1, 2, 5, 15, 30, and 60 min. Plasticizing with ammonia was carried out using a 25% aqueous ammonia solution with longer plasticizing times of 5, 15, 30, and 60 min, 24 h, and 48 h. The different choice of time intervals was based on the different influence of the plasticizing media (water vs. aqueous ammonia solution) on wood moldability.

#### 2.2.4. Pneumatic Method

**Figure 8:**
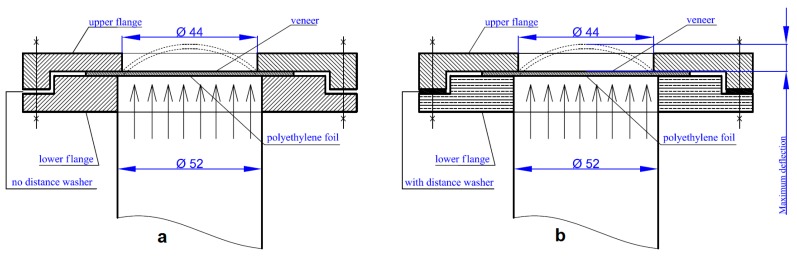
Veneer samples during pneumatic testing: (**a**) Firm holding; (**b**) Gentle holding with a distance spacer (unit: mm).

The final method of 3D molding was the pneumatic method (Figure 8) performed on beech and birch samples. The first group consisted of square samples with the dimensions of 100 mm × 100 mm at moisture of 0%, 8%, 16%, 20%, 30%, and 100% with firm holding. The second group consisted of circular samples with the diameter of 60 mm at moisture of 8%, 20% and 100% with both gentle and firm holding.

All samples were tested in a pneumatic apparatus, using of the principle of loading by compressed air, designed by Professor Ján Zemiar from Technical University in Zvolen, Slovakia, for this research as reported by Zemiar and Fekiač [15]. The force was applied to the sample’s entire surface with an equal specific loading at a given point in time but increasing over time. Air intake was regulated such that veneer cracking occurred within 1.5 min. The 3-D moldability was also evaluated according to the maximum deflection.

The holding system was designed to prevent a possible occurrence of warping at the samples’ circumference similarly as for mechanical method. The holding was ensured using an upper and lower flange between which the sample was inserted. The sample’s surface exposed to air was covered with a polyethylene foil in order to prevent permeation of air through the veneer during the testing. Firm holding was realized using the upper and lower flanges connected to each other without a distance spacer and firmly secured with eccentrics that provided the circumferential pressure (Figure 8a). Gentle holding of samples used aforementioned distance spacer (Figure 8b).

#### 2.2.5. Measurements

The values for maximum loading force were directly downloaded from the data logger onto a computer. The maximum deflection was measured at the center-point of the circle samples or at the intersection of the diagonals of square samples (Figure 6), using an ID-C 543-474B digital indicator (Mitutoyo, Kawasaki, Japan) with a precision of 0.01 mm.

The dimensions of the samples, used for calculating the density, were measured using a 500/150/20 digital caliper (Mitutoyo) with precision of 0.01 mm.

**Figure 9:**
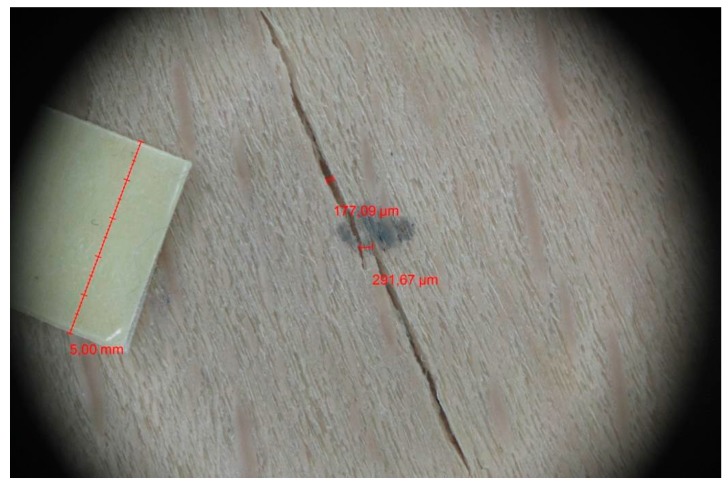
Beech sample magnified 60× with data from X-Loupe Solution software.

Veneer cracks and edge warping created during 3D molding were evaluated only for the first comparison group. These defects were detected using a set consisting of a Canon IXUS 120 IS photo camera (Canon Inc., Tokyo, Japan) and the X-Loupe G20 module (Lumos Technology Co. Ltd., Taipei, Taiwan), then evaluated using X-Loupe Solution software (Lumos Technology Co. Ltd.). This evaluation used a calibration strip with 5 mm dimensions (Figure 9).

## 3. Results and Discussion

### 3.1. Maximum Deflection

#### 3.1.1. General Basic Mechanical Method

**Figure 10:**
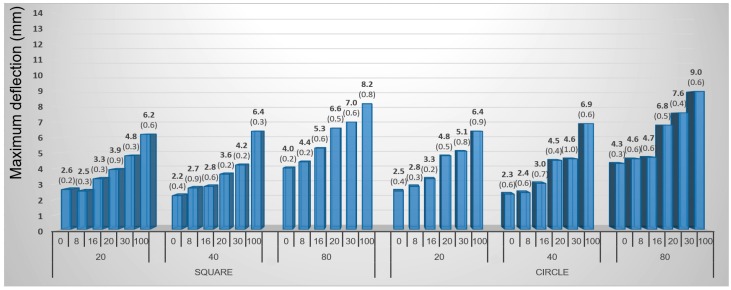
Influence of punch diameter, moisture content, and shape of veneers on maximum deflection of birch veneer during 3D molding by mechanical method. (Note: 0, 8, 16, 20, 30 and 100—moisture content by percent, 20, 40 and 80—diameter of pressing punch in mm).

**Figure 11:**
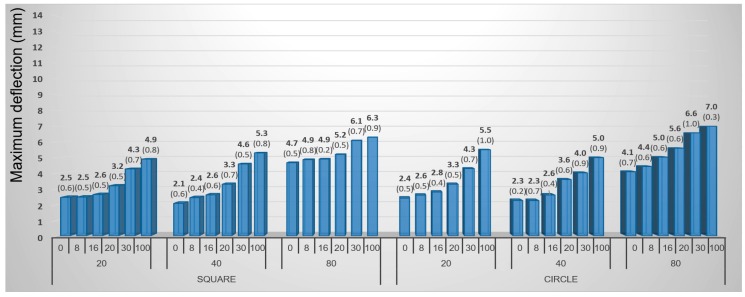
Influence of punch diameter, moisture content, and shape of veneers on maximum deflection of beech veneer during 3D molding by mechanical method. (Note: 0, 8, 16, 20, 30 and 100—moisture content by percent, 20, 40 and 80—diameter of pressing punch in mm).

Birch veneers can be considered generally more suitable material for 3D molding. In all cases, birch samples reached higher maximum deflection values as compared with the beech veneers. The highest values of maximum deflection for birch were measured at around 9 mm (Figure 10), while the beech veneers did not exceed the limit value of 7 mm (Figure 11).

The expected influence of moisture content on veneer deflection values was confirmed. Maximum deflection of veneers increased with increasing moisture in all cases.

The shape of the samples can be considered a factor insignificantly affecting maximum deflection values. Although circle samples achieved slightly higher average value of maximum deflection than the square ones, this difference was quite small. Since the differences were very small, the square shape of the samples is more suitable for the 3D molding process, because this shape is typically achieved in slicing or cutting of veneers and it do not need to be subsequently modified to a circle.

#### 3.1.2. Mechanical Method with Lamination Treatment and Steaming

The effect of punch diameter had proved to be a factor significantly influencing veneer deflection. Maximum deflection values increase with increasing punch diameter. Nevertheless, the consequence of increasing deflection with the increasing punch diameter cannot be directly attributed to the punch diameter but rather to the influence of increasing dimensions of the samples with unchanged thickness.

Wood species had a significant influence on maximum deflection of veneers as in previous case, i.e., higher maximum deflection was measured in birch wood. In general, wood species with uniform width of annual rings, such as birch, can be recommended.

**Figure 12:**
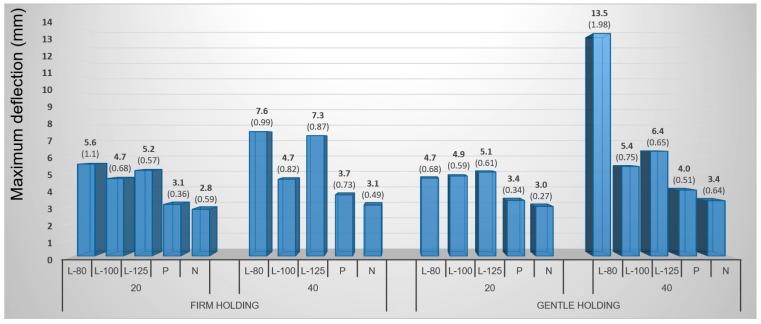
Influence of punch diameter, treatment method, and holding types of veneers on maximum deflection of beech veneer during 3D molding by mechanical method. (Note: L-80, L-100, and L-125—lamination using 80, 100 and 125 μm thick foil, P—plasticizing by steam, N—no treatment, 20 and 40—diameter of pressing punch in μm.)

Steaming is a plasticizing method, which can markedly increase wood bendability. Due to the effects of plasticizing, the average value achieved for maximum deflection of beech samples was 3.6 mm while an average value of 3.1 mm was achieved for untreated samples, which is 14% decline (Figure 12). Maximum deflection values were even higher in birch samples, i.e., 5.1 mm for plasticized samples but an average value of 4.1 mm for untreated samples. These values indicate an increase in value of 24% by plasticizing.

Although the comparison results show a significant effect of plasticizing, however, this effect is insufficient from a practical perspective. Obviously, the effect of metal strap with the end stops in conjunction with the plasticizing, which make possible significantly increase the bendability of wood components, is missing.

**Figure 13:**
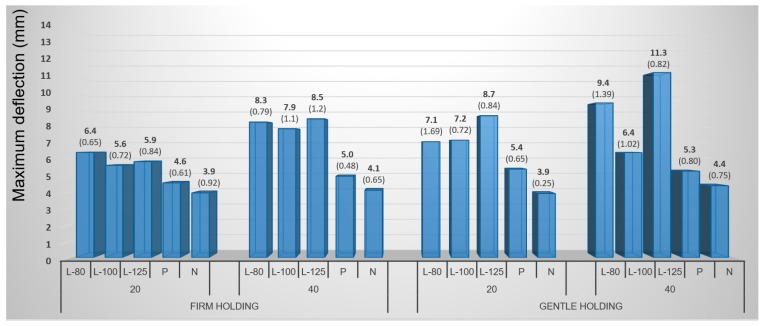
Influence of punch diameter, treatment method, and holding types of veneers on maximum deflection of birch veneer during 3D molding by mechanical method. (Note: L-80, L-100, and L-125—lamination using 80, 100 and 125 μm thick foil, P—plasticizing by steam, N—no treatment, 20 and 40—diameter of pressing punch in mm).

The most important contribution comes in introducing and testing the new and innovative method of veneer treatment using lamination before its 3D molding. A positive influence of the laminating foil can be compared to that of the bending strap. The laminating foil ensures shifting of the neutral axis and thereby forces the sample to deform mainly in pressure, while the tensile stresses are taken over by the foil. Lamination increased the values of maximum deflection of beech veneers from 3.1 mm for untreated samples to the average of 7.9 mm when using the laminating foil with 80-µm thickness—a 155% increase. Laminated birch veneers reached even higher values of maximum deflection than the plasticized ones (Figure 13).

Veneers, laminated with 125 μm-thick foil, averaged the highest values of maximum deflection of 8.6 mm while untreated veneers achieved only 4.1 mm on average. In this case, a 110% increase in deflection was achieved. The laminated veneers with 80 μm-thick foils reached 10.3% lower values (7.9 mm) than those with 125 μm-thick foils. The 100 μm-thick foils had the least influence. However, the dependence of the maximum deflection on increasing the thickness of the foil had no unambiguous character.

Probably, better significant increase in bendability can be reached by combining the wood lamination and plasticizing. However, the solution of the proposed method is still at the stage of technical solutions.

#### 3.1.3. Mechanical Method with Plasticizing by Water and Ammonia

**Figure 14:**
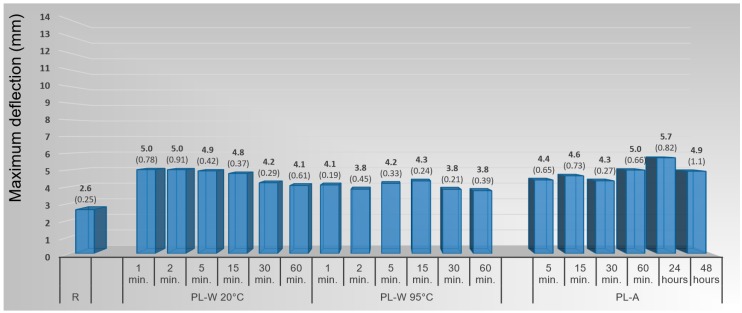
Influence of plasticizing method and plasticizing time on maximum deflection of beech veneers during 3D molding by mechanical method. (Note: PL-W—plasticizing by water, PL-A—plasticizing by ammonia, 20 °C and 95 °C—plasticizing temperatures).

Although plasticizing by soaking in cold water had a positive effect on maximum deflection, the increasing plasticizing time caused a gradual decrease in the effect (Figure 14). The strongest effect of cold water was found for the plasticizing times of 1 and 2 min, when deflection increased by 92% in comparison with the reference (non-plasticized) samples. Paradoxically, worse moldability was found for hot water plasticizing. The greatest maximum deflection was determined with plasticizing time of 15 min, where there occurred a 65% increase in comparison with the untreated samples. Consequently, the dependence of deflection on plasticizing time was ambiguous. In this case, however, the most marked increase in wood moisture content occurred, and this may be the cause behind this observation.

Plasticizing using an aqueous solution of ammonia had only a different character than was the case with the use of cold or hot water. The most effective plasticizer was an aqueous ammonia solution at a time of 24 h because the maximum deflection increased by 119% in comparison with the reference samples.

#### 3.1.4. Pneumatic Method

**Figure 15:**
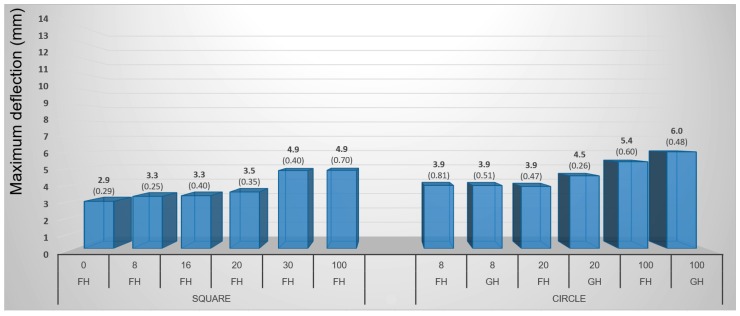
Influence of moisture content, shape of sample, and holding type on maximum deflection of birch samples during 3D molding by pneumatic method. (Note: 0, 8, 16, 20, 30 and 100—moisture content by percent, FH—firm holding, GH—gentle holding).

**Figure 16:**
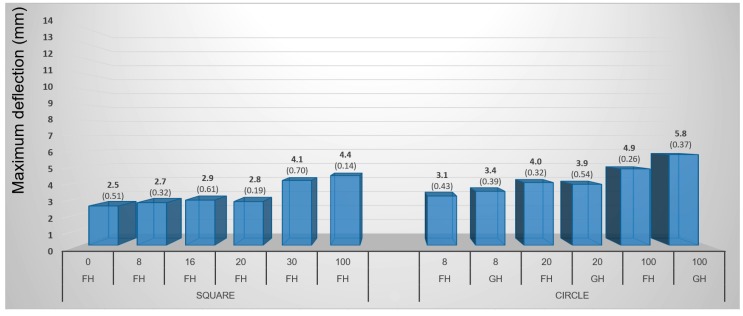
Influence of moisture content, shape of sample, and holding type on maximum deflection of beech samples during 3D molding by pneumatic method. (Note: 0, 8, 16, 20, 30 and 100—moisture content by percent, FH—firm holding, GH—gentle holding).

Higher values of maximum deflection (4.2 mm on average) were found at birch veneers while beech veneers reached only 3.7 mm. The difference was thus 13.5% (regardless of the samples’ shape) (Figure 15 and Figure 16). In general, the maximum deflection of veneer grew proportionally with increasing moisture content in all cases even though the differences between the lower moisture contents of 8%, 16%, and 20% were not very significant. Circular veneers reached 29.4% higher maximum deflection than square veneers did. Square samples are more suitable for practical purposes because they are easier to prepare and use. On the other hand, the circular samples are more suitable for testing because each point of their circumference is equidistant from the center and that ensures an equal distribution of force to the surface during molding.

Circular-shaped samples, with gentle holding (GH), had the highest average maximum deflection of 4.6 mm. Veneers with firm holding (FH) reached 9.5% lower deflection values (i.e., 4.2 mm).

For pneumatic molding, the most suitable combination can be defined as follows: birch veneers with a circular shape, 100% moisture content and gently held.

**Figure 17:**
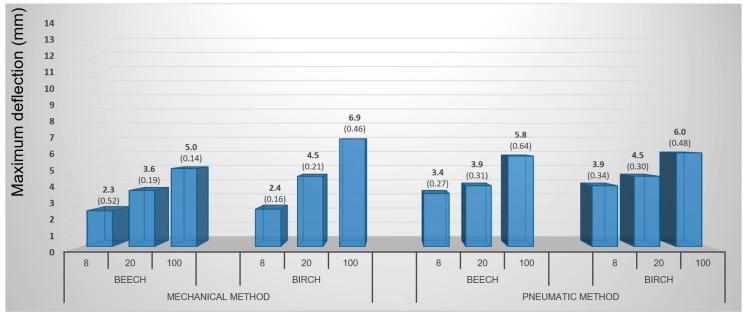
Comparison of the general mechanical and pneumatic methods of 3D molding of circular veneer samples with gentle holding. (Note: 8, 20, and 100—moisture content in percent).

The results of 3D molding using the pneumatic and mechanical methods are best comparable on basis of such common parameters as the samples’ circular shape and gentle holding. Punch diameter of 40 mm is the most suitable because the internal opening for molding in both cases was 44 mm (see Figure 8). Figure 17 shows that the maximum deflection was equally dependent upon the individual factors. In both cases, birch veneers achieved the best results. The influences of moisture content as well as veneer shape had also a similar character. Beech veneers achieved higher maximum deflection values in the pneumatic method while birch veneers had higher values in mechanical method. The pneumatic method is specific in having markedly smaller differences due to the individual moisture contents.

### 3.2. Warping and Micro Cracks

**Figure 18:**
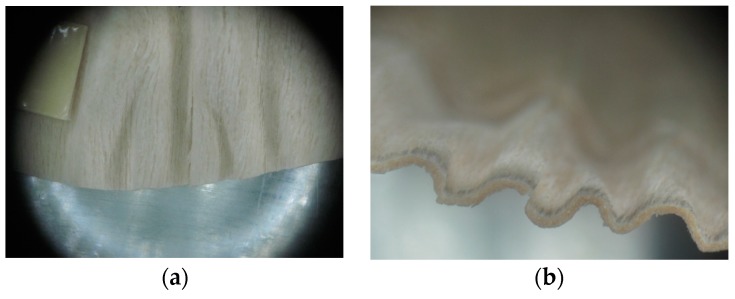
An example of warped edges on birch veneers: (**a**) Top view at sample; (**b**) Side view at sample.

Warping of the veneer edges occurred only at 24 samples. All these samples had been clamped with gentle holding whereby the veneer was able to move and therefore to warp. The warping started to occur at maximum deflection of more than 5 mm. In certain cases, birch samples had edge cracks but these were not very long (Figure 18).

**Figure 19:**
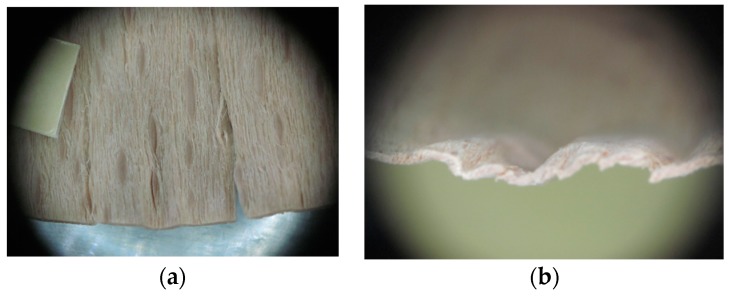
An example of warped edges on beech veneer: (**a**) Top view at sample; (**b**) Side view at sample.

Edge warping of beech samples was accompanied by larger and longer cracks (Figure 19). The weakest connections between wood cells are at the edges where rays are joined with the surrounding wood, therefore cracks occur in these places, and that is apparent in the beech veneers.

Micro-cracks were analyzed in photographs of veneers after their warping. The X-Loupe Solution software measured the width of the micro-cracks as well as the distance between them. Characteristic types of micro-cracks occurring within the groups of samples for two punch diameters are shown at Table 4 and Table 5.

## 4. Conclusions

The results did not confirm significant differences in the maximum deflection values between the mechanical and pneumatic method. The method of 3D molding is closely related to the wood species and its properties. In general, birch veneers reached higher maximum deflection than beech ones in using both methods.

The best moldability in the mechanical method was provided using veneer lamination, achieving better results than in steam plasticizing. The highest maximum deflection was at veneers using foils with 80 and 125 μm thicknesses. The influence of holding during molding was ambiguous even though a slightly higher maximum deflection was found out in the case of gentle holding of the veneers.

Plasticizing with a 25% ammonia solution, in the mechanical method, provided better results than plasticizing with cold and hot water. With increasing plasticizing time, the maximum deflection achieved gradually increase. On the other hand, maximum deflection paradoxically decreased with increasing plasticizing time when using cold water. During plasticizing with hot water there was a slight increase of maximum deflection with increasing plasticizing time but only until the 15 min time period. Thereafter, the values began to decrease.

The pneumatic method provided smaller differences in the maximum deflection values between the individual moisture groups. Higher values were recorded, if used gentle holding.

Micro-cracks occurred in both wood species. Micro-cracks at beech veneers were wider and longer. Edge warping occurred only with gentle holding of veneers because the wood could deform due to molding.

## Figures and Tables

**Table 1:** Relationship between the dimensions of pressing punches and samples.

Diameter of Punch (mm)	Diameter of Circular Samples (mm)	Dimensions of Square Samples (mm)
20	ø 60	60 × 60
40	ø 80	80 × 80
80	ø 100	100 × 100

**Table 2:** Conditioning parameters.

Parameter	Equilibrium Moisture Content					
	0%	8%	16%	20%	30%	100%
Air moisture (%)	0	40	78	87	96	soaking in water
Temperature (°C)	103 ± 2	20				

**Table 3:** Mechanical and pneumatic methods in relation to conditions and factors.

Method Condition	Mechanical							Pneumatic	
	General Basic Mechanical Method	I. Comparison Group			II. Comparison Group			General Basic Pneumatic Method	
		No Treatment	Lamination	Plasticizing by Steam	No Treatment	Plasticizing by Water	Plasticizing by Ammonia		
Wood species	European beech	European beech	European beech	European beech	European beech	European beech	European beech	European beech	European beech
	Silver birch	Silver birch	Silver birch	Silver birch				Silver birch	Silver birch
Moisture content	0%	16%	16%	16%	16%	16%	16%	0%	8%
	8%							8%	
	16%							16%	20%
	20%							20%	
	30%							30%	100%
	100%							100%	
Shape of sample	Square	Circle	Circle	Circle	Circle	Circle	Circle	Square	Circle
	Circle								
Diameter of pressing punch	20 mm	20 mm	20 mm	20 mm	40 mm	40 mm	40 mm	-	-
	40 mm									
		40 mm	40 mm	40 mm					
	80 mm								
Thickness of lamination foil	-	-	80 µm	-	-	-	-	-	-
			100 µm						
			125 µm						
Type of sample fixation	gentle holding	firm holding	firm holding	firm holding	firm holding	firm holding	firm holding	firm holding	firm holding
		gentle holding	gentle holding	gentle holding					gentle holding
Water/steam temperature	-	-	-	95 °C	-	20 °C	20 °C	-	-
						95 °C			
Plasticizing time	-	-	-	15 min.	-	1 min	5 min	-	-
						2 min	15 min		
						5 min	30 min		
						15 min	60 min		
						30 min	24 h		
						60 min	48 h		
Crack and warping evaluation	No	Yes	Yes	Yes	No	No	No	No	No
Number of samples *	720	80	240	80	10	120	60	120	120

* Note: 10 samples per combination of factors/conditions.

**Table 4:** Micro-cracks of the I. comparison group using a punch with 20 mm diameter.

Treatment Type	Firm Holding		Gentle Holding	
	Beech	Birch	Beech	Birch
L-80	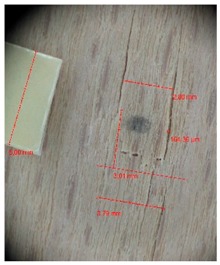	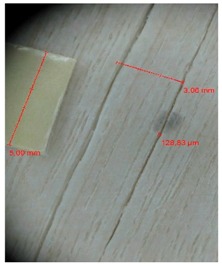	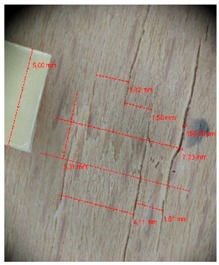	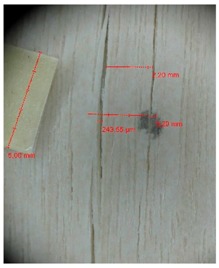
L-100	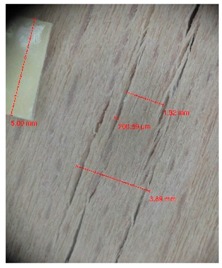	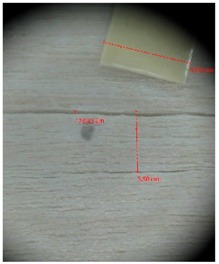	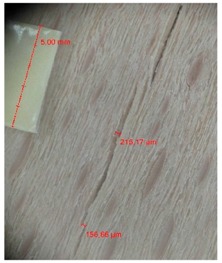	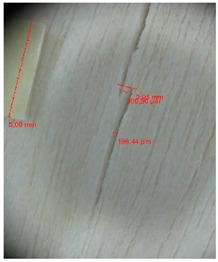
L-125	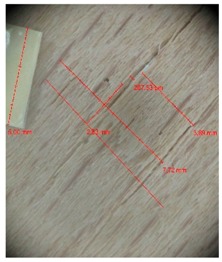	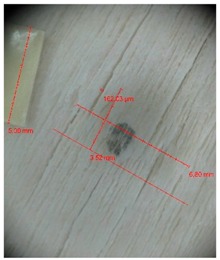	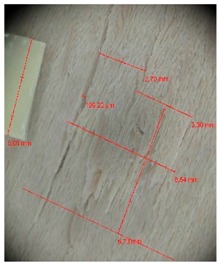	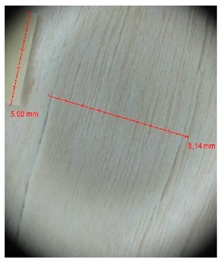
P	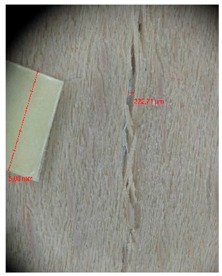	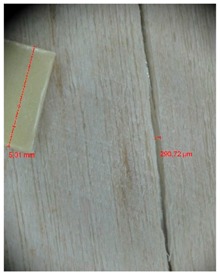	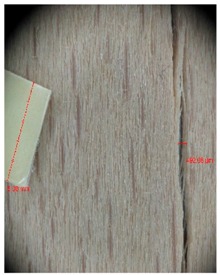	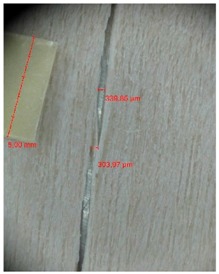
N	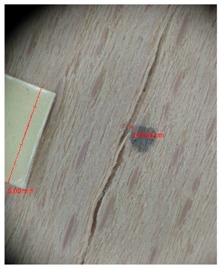	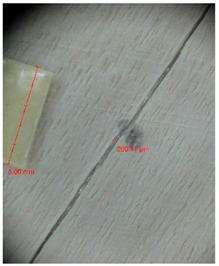	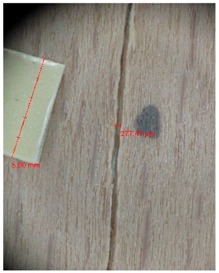	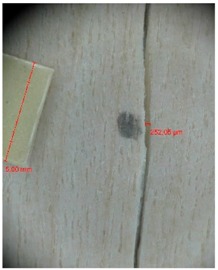

Note: P—plasticized by steam, N—no treatment, L-80, L-100, and L-125—lamination using foil 80, 100 and 125 μm thick.

**Table 5:** Micro-cracks of the I. comparison group using a punch with 40 mm diameter.

Treatment Type	Firm Holding		Gentle Holding	
	Beech	Birch	Beech	Birch
L-80	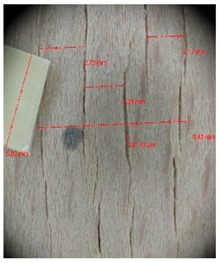	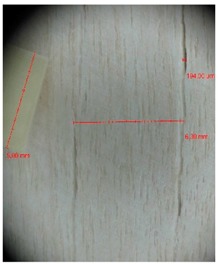	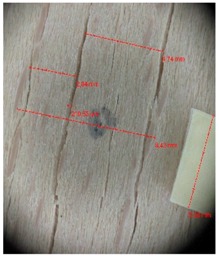	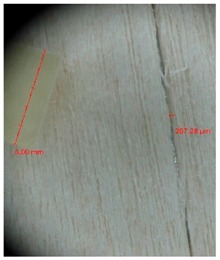
L-100	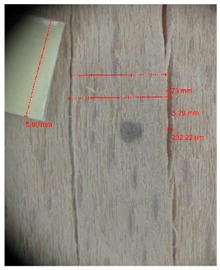	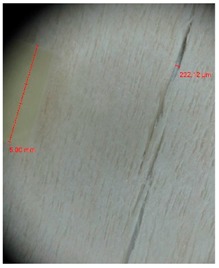	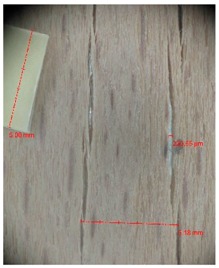	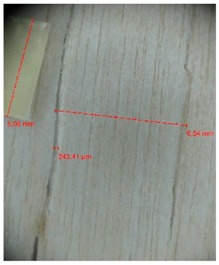
L-125	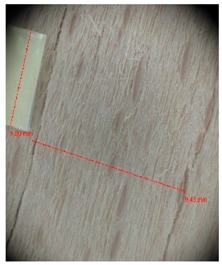	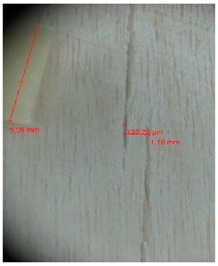	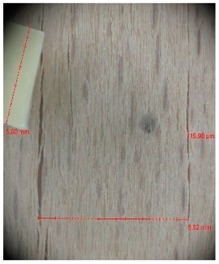	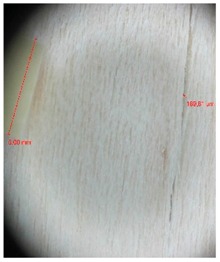
**P**	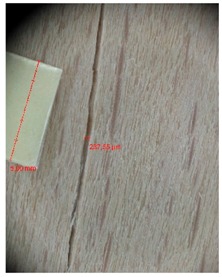	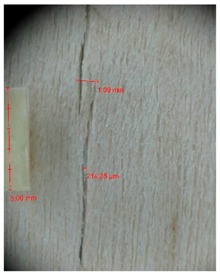	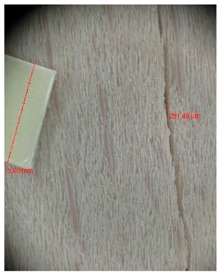	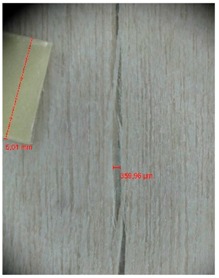
**N**	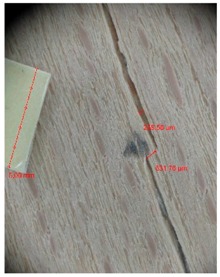	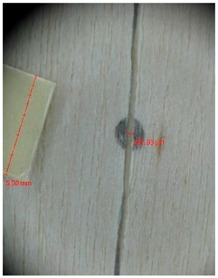	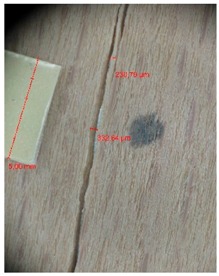	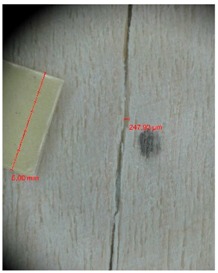

Note: P—plasticized by steam, N—no treatment, L-80, L-100, and L-125—lamination using foil 80, 100 and 125 μm thick.
